# Beyond gluten-free diet: a critical perspective on phase 2 trials on non-dietary pharmacological therapies for coeliac disease

**DOI:** 10.3389/fnut.2024.1501817

**Published:** 2025-01-07

**Authors:** Davide Scalvini, Chiara Scarcella, Giulia Mantica, Erica Bartolotta, Stiliano Maimaris, Erica Fazzino, Federico Biagi, Annalisa Schiepatti

**Affiliations:** ^1^Department of Internal Medicine and Therapeutics, University of Pavia, Pavia, Italy; ^2^Istituti Clinici Scientifici Maugeri IRCCS, Gastroenterology Unit of Pavia Institute, Pavia, Italy

**Keywords:** coeliac disease, gluten-free diet, alternative therapies, pharmacological therapies, persistent symptom

## Abstract

Coeliac disease is an immune-mediated chronic enteropathy, with a prevalence of around 1% in the general population and occurring in genetically susceptible individuals after the ingestion of gluten proteins present in wheat, rye and barley. Currently, a strict lifelong gluten-free diet is the cornerstone of treatment of coeliac disease. However, maintaining strict dietary adherence is challenging for many patients, due to the high costs, the highly restrictive nature of the diet and the impact on patients’ quality of life. Moreover, a tiny minority of coeliac patients can develop pre-malignant/malignant complications of coeliac disease, a group of conditions, that despite being rare, are still burdened by a poor prognosis due to the lack of effective therapies. Therefore, the development of pharmacological treatments as an alternative to or supportive of a gluten-free diet is still an unmet need. The identification of new pathogenetic targets in the last years has enabled the development of several candidates molecules, many of which have been investigated in phase 2/3 clinical trials. In this narrative review we aim to summarise the investigational therapies that have been evaluated in phase 2/3 trials and provide a critical overview on the latest advances in this field.

## 1 Introduction

Coeliac disease (CeD) is a chronic immune-mediated enteropathy developing in genetically susceptible individuals after the ingestion of gluten (1–4). CeD is characterised by a prevalence of around 1% in the general population, a very heterogeneous clinical picture and an increased mortality compared to the general population, predominantly due to the development of pre-malignant and malignant complications such as refractory CeD, abdominal lymphomas and small-bowel adenocarcinoma (1–6). A strict lifelong gluten-free diet (GFD) is the cornerstone of treatment for CeD, leading to resolution of symptoms and small bowel lesions in the vast majority of patients (1–4). However, great interest has been devoted to alternative/supportive therapies for several reasons (Table 1).

**Table 1:** Reasons for needing alternative/supportive therapies to a gluten-free diet.

Persistence of symptoms/histological lesions despite a GFD
Lack of effective therapies for refractory and complicated CeD
Inadvertent gluten ingestion
Availability of GF foodstuffs and other barriers to long-term adherence (economical, social e.g.)
Palatability of GF foodstuffs
Individual super-sensitivity to gluten

GFD, gluten-free diet; CeD, coeliac disease; GF, gluten-free.

Firstly, a GFD can be demanding for many patients to maintain due to psychological, economic and social barriers (7–10), and in addition to this, many patients also experience persistent symptoms despite a GFD (11–13). Persistence of symptoms despite a GFD is a common and relevant clinical scenario, that can affect up to 30–50% of coeliac patients and be due to many different underlying etiologies, either related or unrelated to CeD itself, and with significant variability in terms of clinical severity (11–13). In some cases, unsatisfactory response to a GFD can be due to development of malignant complications of CeD, which, although rare, are burdened by a very dismal prognosis and for which, currently, no standardised and curative treatments are available (14–16). Patients can also experience persistent symptoms due to voluntary or involuntary transgressions to a GFD, or because some of them have been reported to be supersensitive to gluten (11–13).

The dissatisfaction of many coeliac patients with a GFD (17) and their interest regarding the possibility of novel therapies (18), put together with recent developments into the underlying pathogenetic mechanisms of CeD (19) have provided ample fuel for research aiming to develop alternative or supportive non-dietary treatments for CeD.

This review aims to provide a state-of-the-art summary and a critical overview on the different types of non-dietary therapies for CeD that have been proposed and evaluated in phase 2/3 clinical trials so far.

## 2 Criteria for literature search

We performed a systematic search of the literature on experimental non-dietary therapies for CeD using the PubMed and Embase databases. The search was conducted on January 17, 2024 using search strings designed to identify relevant phase 2/3 trials focussing on CeD and its non-dietary treatments, including pharmacological and other experimental therapies. Only full-text papers were considered for inclusion. No temporal or language restrictions were applied to the search. The search terms encompassed various synonyms and keywords related to CeD and therapeutic approaches to ensure a broad coverage of the existing literature. The exact search strings used for each database are listed below:

- PubMed:

(celiac disease[mesh] OR coeliac disease[title] OR celiac disease[title] OR celiac disease[ot] OR coeliac disease[ot] OR gluten sensitive enteropathy[title]) AND (treatment[title/abstract] OR drug[title/abstract] OR pharmacological[title/abstract] OR trial[title/abstract])

- Embase:

(‘celiac disease’:ti,kw OR ‘coeliac disease’:ti,kw OR ‘gluten sensitive enteropathy’:ti,kw) AND (‘treatment’:ti,ab,kw OR ‘drug’:ti,ab,kw OR ‘pharmacological’:ti,ab,kw OR ‘trial’:ti,ab,kw)

Search results from both databases were then merged, and after removing duplicates, we screened the titles and abstracts of the retrieved articles to identify relevant studies. Additionally, we reviewed the reference lists of selected articles and reviews to identify any additional relevant studies that may not have been captured by our initial search.

**Figure 1:**
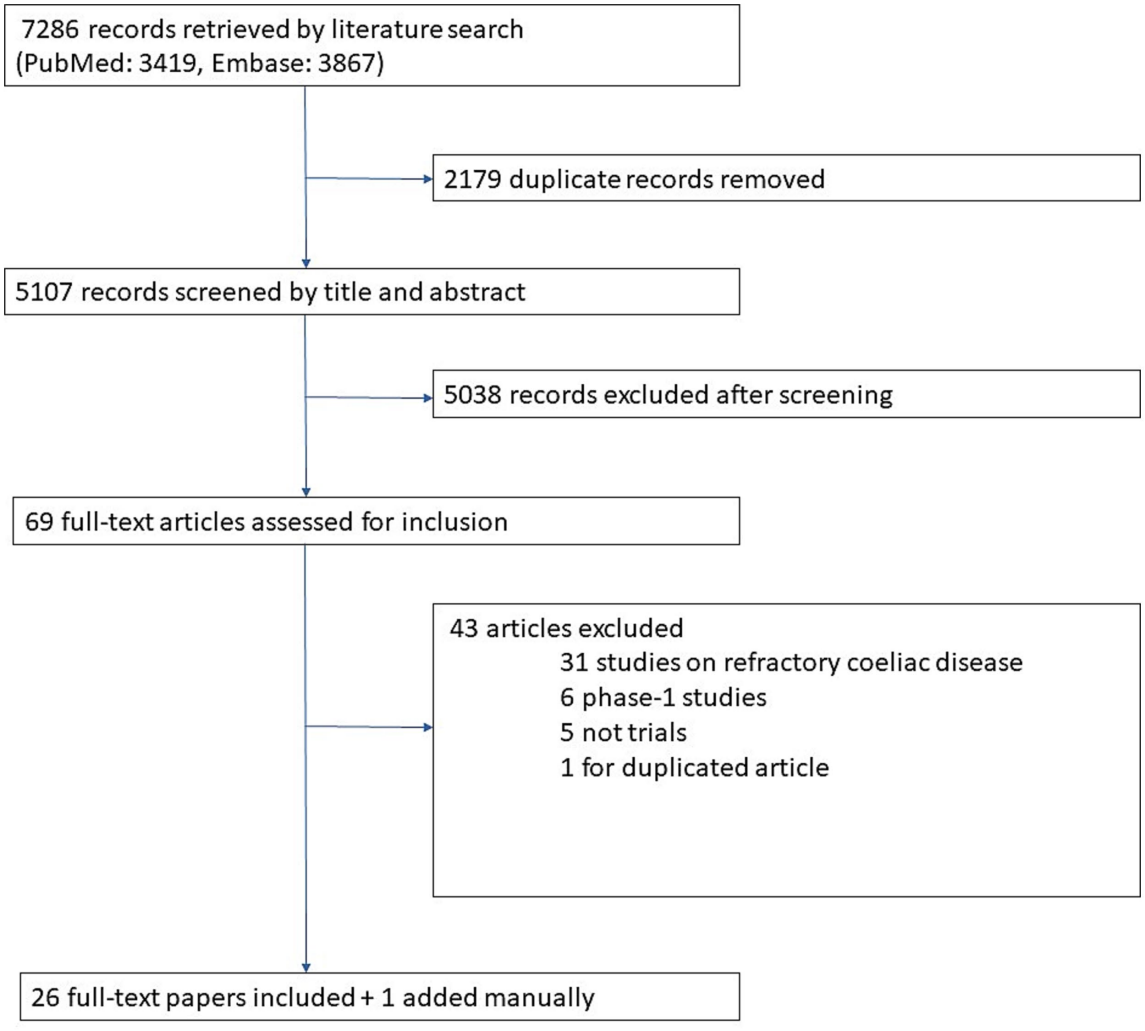
Flowchart of studies included the review.

The flowchart in Figure 1 illustrates the results of our literature search and our screening process for identifying eligible articles. Overall, 7,286 records were retrieved by our literature search. After removing duplicates and screening titles and abstracts, 69 papers were considered for inclusion. After full-text review 26 of them were included. Finally, 1 additional paper published after our literature search was also included, so 27 papers were included overall.

## 3 From pathophysiology of coeliac disease to therapeutic targets

**Figure 2:**
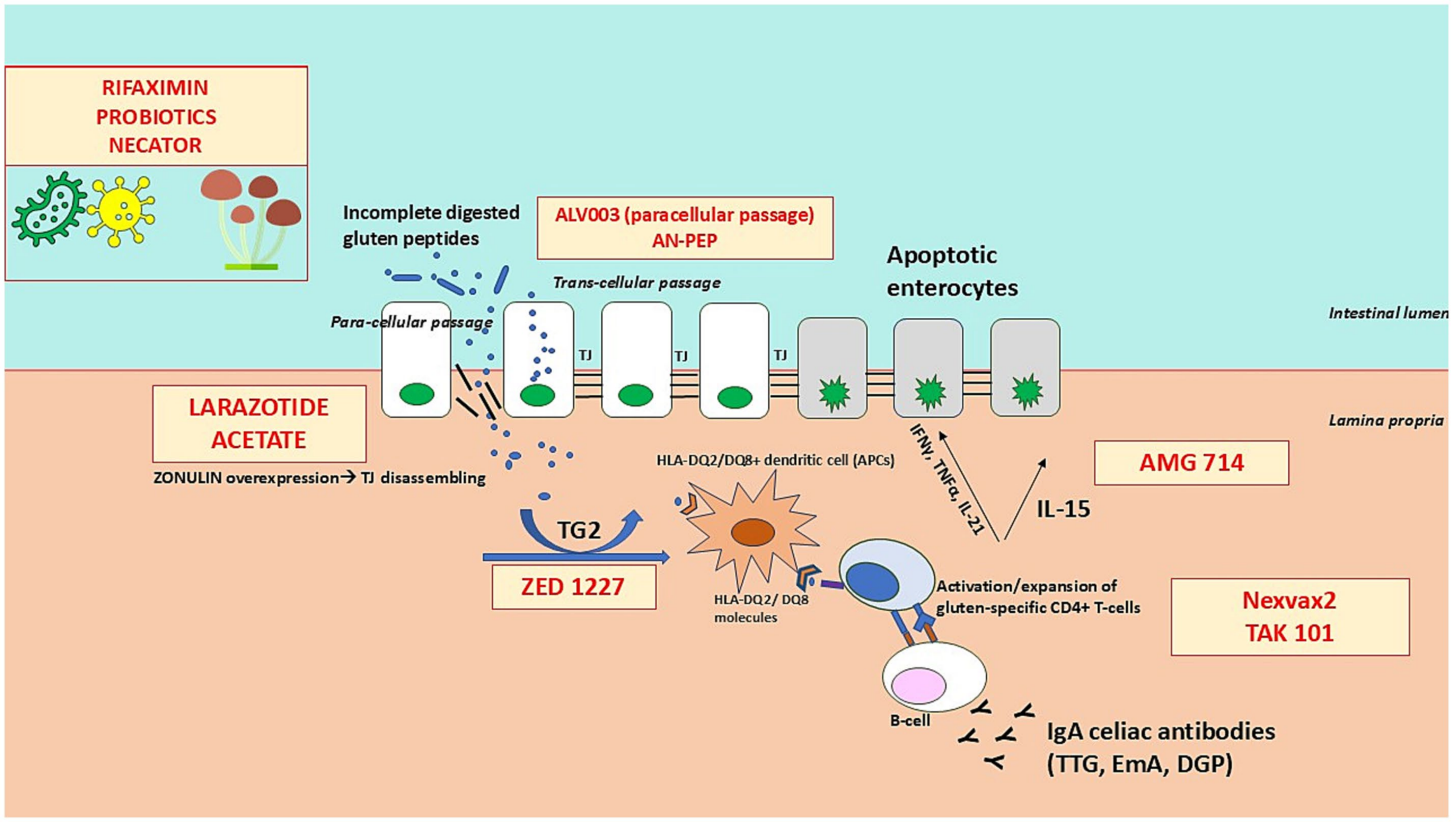
Therapeutic targets and mechanisms of action in coeliac disease. The figure illustrates the key steps in the pathogenesis of coeliac disease and highlights where current investigational therapies intervene. These therapies target different underlying mechanisms, including: (1) Glutenases (e.g., ALV003, AN-PEP) that enzymatically degrade immunogenic gluten peptides in the gastrointestinal lumen to prevent immune activation; (2) Intestinal barrier modulators (e.g., larazotide acetate) that enhance tight junction function to reduce intestinal permeability and prevent translocation of gluten peptides; (3) Tissue transglutaminase-2 (TG2) inhibitors (e.g., ZED1227) that block the deamidation of gluten peptides, reducing their immunogenicity; (4) Immunotherapies (e.g., Nexvax2, TAK-101) that aim to induce immune tolerance by modulating gluten-specific T-cell responses; and (5) Immunomodulators targeting pro-inflammatory cytokines (e.g., AMG 714, an anti-IL-15 antibody) to suppress immune-mediated intestinal inflammation. By disrupting various stages of the immune response to gluten, these therapies offer potential alternative or adjunctive treatments to the gluten-free diet in coeliac disease.

A thorough description of the pathophysiology of CeD is beyond the scope of this review. However, we would like to provide the readers with a description of the different molecules tested so far, which have been classified according to their mechanisms of action and their specific pathogenetic targets. This is illustrated and briefly described in Figure 2.

## 4 Peptidases to digest gluten

Gluten owes its immunogenicity to its high content of proline and glutamine, which are not efficiently degraded by the enzymes of the gastrointestinal tract. Consequently, these proteins are capable of triggering the immune response in individuals with CeD.

The degradation of gliadin peptides at the level of the stomach/intestinal lumen before they reach the *lamina propria* aims at preventing the activation of the immune cascade leading to the intestinal damage. This therapeutic approach is usually based on the use of peptidases able to degrade gluten through their proteolytic action, usually identified as to glutenases. Table 2 summarises the phase 2 trials on glutenases. Several trials have been conducted evaluating 2 different types of endopeptidases, namely ALV003 and AN-PEP (20–28), which are described below.

**Table 2:** Phase-2 studies on gluten digestive endopeptidase.

Study	Type	Molecule	Population	Endpoints (primary; secondary)	Gluten challenge	Adverse events	Key results
Tye-Din et al. (21)	Double-blind RCT	ALV003	Adult CeD on GFD >8 weeks, HLA DQ2+	IFN-*γ* ELISpot responses; symptom response and antibody levels after GC	16 g/day for 3 days	Nausea, bloating, abdominal pain (the only one more frequent in intervention group)	ALV003 pre-treatment abolished immune responses but not symptoms
Lähdeaho et al. (22)	Double-blind RCT	ALV003	CeD on GFD >1 year, in remission, TG2-IgA negative	Vh:Cd ratio, IEL densities, serologic markers, symptoms, QOL	2 g/day for 6 weeks	Nausea, vomiting, abdominal pain (no differences between groups)	ALV003 prevented significant mucosal deterioration
Murray et al. (23)	Double-blind RCT, dose-ranging	ALV003	CeD on GFD >1 year, with GI symptoms	Change in Vh:Cd; IELs, serology, symptoms, safety	None	Bloating, nausea, abdominal pain (no differences between groups except for one moderate episode of fungal infection attributable to the treatment)	No improvement of histology and symptom scores compared with placebo; significant improvements in histology and symptom scores in all groups
Syage et al. (25)	Double-blind RCT, dose-ranging	ALV003	Seropositive and seronegative CeD, Vh:Cd ≤2.0	Symptoms, QOL, serology	None	No serious AEs reported	Dose-dependent improvement in symptoms and QOL for seropositive patients
Murray et al. (24)	Double-blind RCT	ALV003	CeD on GFD >1 year, Vh:Cd >2, TTG negative	Prevention of mucosal damage; Symptoms, IELs	2 g/day for 6 weeks	Nausea, bloating, Diarrhoea (no differences between groups)	Reduced gluten-induced intestinal mucosal damage and symptom severity
Tack et al. (27)	Double-blind RCT pilot study	AN-PEP	CeD on GFD >1 year, Marsh 0 or I, TTG and EMA negative	Safety and efficacy with gluten challenge	7 g/day for 2 weeks	No serious AEs; mild and transient gastrointestinal complaints	Well tolerated; primary endpoint not met due to lack of clinical deterioration upon placebo
Stefanolo et al. (28)	Double-blind RCT, exploratory	AN-PEP	CeD on GFD >2 years	Stool GIP; CSI, CeD-specific serology, QOL	Inadvertent gluten exposure	No major AEs reported.	AN-PEP did not reduce overall GIP stool excretion, but lowered prevalence of severe symptoms vs. placebo

RCT, Randomised Controlled Trial; CeD, Coeliac Disease; GFD, Gluten-Free Diet; Vh:Cd, Villus height:Crypt depth ratio; IEL, Intraepithelial Lymphocytes; QOL, Quality of Life; TTG, Tissue Transglutaminase; EMA, Endomysial Antibodies; GIP, Gluten Immunogenic Peptides; GC, gluten challenge; CSI, Coeliac Symptom Index; AEs, adverse events.

### 4.1 ALV003 (latiglutenase)

ALV003, also known as latiglutenase, is the most commonly studied glutenase. ALV003 is a glutenase composed of two gluten-specific proteases: ALV001 and ALV002. ALV001 is a genetically engineered form of cystine endoprotease B, isoform 2 sourced from barley (*Hordeum vulgare*) while ALV002 is a modified form of prolyl endopeptidase extracted from the bacterium *Sphingomonas capsulata*. As of today, ALV003 is being studied through phase 2 trials as the molecule has progressed beyond phase 1 trials, demonstrating tolerability and safety (20).

In a randomised, double-blind study, the efficacy of ALV003 was evaluated by pre-treating food with this glutenase and assessing the T-cell response in 20 patients (including 10 treated with a placebo). Unlike the food pre-treated with ALV003, 6 out of 10 patients in the placebo-treated gluten group exhibited gluten-specific T-cell response in peripheral blood. Both groups, however, experienced gastrointestinal symptoms after ingestion. It is worth noting that, in contrast to other studies investigating ALV003 where a dose of 2 g of gluten for 6 weeks was administered (22, 24), this one involved the administration of 16 g (21).

In a randomised, placebo-controlled, double-blind clinical phase 2 trial by Lähdeaho et al., ALV003 appeared to mitigate gluten-induced damage to the small intestinal mucosa in patients with CeD, within the context of a daily gluten-free diet containing up to 2 g of gluten for 6 weeks, although a statistically significant difference in the presence of any symptoms was not found (22). Conversely, Murray et al. in a multicenter, randomised, double-blind, placebo controlled, dose-ranging study enrolled 494 symptomatic coeliac patients on a GFD for at least 1 year with duodenal mucosal atrophy to assess the efficacy and safety of ALV003. The primary endpoint evaluated any histological changes in the mucosa, while the secondary endpoints included the number of intraepithelial lymphocytes (IELs), antibody positivity, symptom frequency, and drug safety. Although the drug was well tolerated by all participants, the study’s endpoints were not achieved, as ALV003 failed to improve villous atrophy or reduce the severity and frequency of symptoms (23).

In a subsequent study including 50 patients receiving 2 g/day of gluten for 6 weeks and 1,200 mg of latiglutenase a reduction in both mucosal damage and symptom severity compared to placebo, was demonstrated (24).

In the ALV003–1221 clinical trial, a multi-center, randomised, double-blind, placebo-controlled study, although the primary endpoint to achieve histological improvement was not met, treated subjects experienced significant improvement in symptoms and quality of life (QOL). There was a statistically significant, dose-dependent reduction in the severity and frequency of symptoms (abdominal pain, bloating, tiredness, and constipation) in subjects treated with ALV003. Interestingly, Diarrhoea and nausea were the only symptoms which did not improve after receiving the glutenase (25).

Overall, ALV003 (latiglutenase) shows mixed prospects. While it demonstrated some efficacy in symptom reduction, inconsistent results across trials and failure to meet primary endpoints in larger studies suggest limited future development potential.

### 4.2 AN-PEP

An endoprotease derived from Aspergillus Niger named AN-PEP is able to degrade both whole gluten and gluten peptides into non-immunogenic residues within minutes (26). A total of 2 studies have evaluated AN-PEP so far (27, 28). Both these studies had limitations due to the small sample size and the short duration of gluten intake (2 weeks). In a randomised double-blind placebo-controlled pilot study, the safety and efficacy of AN-PEP were evaluated. However, prevention of histological damage after receiving gluten and AN-PEP, i.e., the primary endpoint, was not met, despite the overall good tolerability by all participants (27).

A recent RCT investigated the role of AN-PEP in reducing stool gluten immunogenic peptides (GIP). While the use of AN-PEP has been associated with a lower incidence of severe GI symptoms, it failed to meet the primary endpoint, as a significant decrease of stool GIP was not found in patients receiving AN-PEP when compared with the placebo group (28).

The presence of nausea, bloating and abdominal pain were the most commonly reported adverse events (AEs) during the administration of gluten-digestive peptidases; however, their incidence rates did not statistically differ from the placebo group.

To summarise, future development for AN-PEP appears limited due to its failure to significantly reduce GIP or prevent histological damage without major modifications to improve its efficacy.

## 5 Intestinal barrier modulators

The intestinal barrier, including its epithelial integrity and tight junctions, is obviously crucial in CeD (29). Tight junctions appear to play a particularly important role in CeD by maintaining intestinal barrier integrity. The main components of tight junctions include occludins, claudins, junctional adhesion molecules (JAM), and zonulin. After gluten exposure, epithelial cell rearrangement and loss of barrier integrity are observed, causing an inappropriate immune response to environmental antigens like gluten (29, 30). These observations prompted many researchers to conduct studies evaluating barrier modulators as alternative therapies to GFD.

### 5.1 Larazotide acetate

Four phase-2 studies have examined larazotide acetate, also known as AT-1001, an 8-amino-acid synthetic peptide able to decrease the intestinal permeability, by acting as an antagonist of the zonulin, a key protein in regulation of the gut’s tight junctions. Larazotide acetate is a paracellular permeability inhibitor derived from a protein produced by *Vibrio Cholerae* and it regulates tight junctions, preventing the passage of gluten into the mucosal *lamina propria* and the subsequent trigger of the inflammatory response. This drug has no effect on the transcellular passage of gluten (31, 32). Table 3 summarise the results of these studies.

**Table 3:** Phase-II studies on Larazotide.

Study	Type	Molecule	Population	Endpoints (primary; secondary)	Gluten challenge	Adverse events	Key results
Paterson et al. (31)	Double-blind RCT	AT-1001	Adult CeD on GFD >6 months, anti-tTG ≤10 EU	Intestinal permeability (LAMA ratio); GI discomfort, AEs, global outcomes, urinary nitrites/nitrates, PBMC markers, cytokine levels	2.5 g for 3 days	Diarrhoes, abdominal discomfort and flatulence. Gastrointestinal symptoms were more frequently detected in the placebo group.	No permeability increases in AT-1001 group; 70% increase in placebo. Fewer GI symptoms in AT-1001 group.
Leffler et al. (57)	Double-blind RCT, dose-ranging	Larazotide Acetate	Adult CeD on GFD ≥6 months, in remission	Intestinal permeability (LAMA ratio); Clinical symptoms (GSRS and CeD GSRS), QOL measures, TTG levels	2.4 g/day for 14 days	Common AEs included headache and UTI.	No difference in LAMA ratios. 0.25 and 4.0 mg doses prevented worsening of GI symptoms vs. placebo.
Kelly et al. (32)	Double-blind RCT, exploratory	Larazotide Acetate	Adult CeD on GFD >6 months, anti-tTG ≤10 EU	Intestinal permeability (LAMA ratio); Clinical symptoms (GSRS), TTG levels	2.7 g/day for 6 weeks	Common AEs included gastrointestinal disorders, fatigue, headache with similar rates between groups	No difference in LAMA ratios. Larazotide reduced symptoms and anti-TTG levels vs. placebo. Similar AEs.
Leffler et al. (56)	Double-blind RCT	Larazotide Acetate	Adult CeD on GFD ≥12 months, anti-tTG IgA <4	Clinical symptoms (weekly CeD-GSRS); Change from baseline in CeD-GSRS, CeD PRO GI and Abdominal domain scores	None	No drug-related serious AEs	0.5 mg dose reduced symptoms vs. placebo. 1 and 2 mg doses no different from placebo. Safety comparable to placebo.

RCT, Randomised Controlled Trial; CeD, Coeliac Disease; GFD, Gluten-Free Diet; LAMA, Lactulose-to-Mannitol; TTG, Tissue Transglutaminase; GI, Gastrointestinal; GSRS, Gastrointestinal Symptom Rating Scale; PBMC, Peripheral Blood Mononuclear Cell; QOL, Quality of Life; PRO, Patient-Reported Outcome; AEs, Adverse Events.

A recent meta-analysis of RCTs on larazotide acetate including 626 CeD patients who underwent ingestion of gluten ranging from 2.5 grams for 3 days up to 2.7 grams for 6 weeks, of which 456 receiving Larazotide acetate and 161 receiving a placebo, showed that the drug reduced the weekly number of symptomatic days and improved symptom severity scores compared to the placebo in patients undergoing gluten challenge. Unfortunately, it failed to demonstrate a reduction in intestinal permeability compared to placebo (33).

During treatment with larazotide acetate, no severe AEs were reported. As previously mentioned, it was able to significantly reduce the incidence of Diarrhoea, abdominal pain, and bloating, which were the most frequently AEs in both the intervention and control groups.

Despite Larazotide acetate’s ability to alleviate gastrointestinal symptoms, it seems unlikely to be a definitive cure for coeliac patients. Instead, it may be considered a complementary option to a GFD in patients with persistent symptoms rather than a substitute of GFD itself.

So far, larazotide investigations have been discontinued despite showing some promise in symptom management.

## 6 Modulators of the immune response to gluten

The third possible strategy to achieve gluten tolerance is to apply drugs that modulate the immune response to gluten and gluten-related peptides (34).

Several molecules and mechanisms have been investigated trying to block different pathways in CeD pathogenesis, as illustrated in Figure 2 and summarised in Table 4.

**Table 4:** Phase-2 studies on pharmacological modulators of the immune response to gluten and transglutaminase 2 inhibitor.

Study	Type	Molecule	Mechanism	Population	Endpoints (primary; secondary)	Gluten challenge	Adverse events	Key results
Goel et al. (35)	Double-blind RCT	Nexvax2	Gluten peptide-based antigen-specific immunotherapy	HLA-DQ2.5+ CeD, 18–70 years, on GFD	AEs; Safety, tolerability, duodenal histology, antibodies, IGRA	9 g/day on days 1–3 and on days 8–10 as cookies	Nausea, vomiting, abdominal pain were more frequent in Nexvax2 group	More AEs with Nexvax2. No significant differences in other endpoints.
Truitt et al. (36)	Double-blind RCT	Nexvax2	Gluten peptide-based antigen-specific immunotherapy	HLA-DQ2.5+ CeD, 18–70 years, GFD >12 months	Safety, tolerability, bioavailability; Pharmacokinetics	Yes	Headache, abdominal distension, nausea were more frequent in Nexvax2 group	Subcutaneous dosing safe, well-tolerated. Higher exposure than intradermal.
Tye-Din et al. (37)	Double-blind RCT	Nexvax2	Gluten peptide-based antigen-specific immunotherapy	HLA-DQ2.5+ CeD, 18–70 years, GFD >12 months	CeD symptoms post-gluten; Blood IL-2, individual symptoms	10 g bolus vital gluten	Nausea, Diarrhoea abdominal pain (no differences between groups)	Nexvax2 did not reduce gluten-induced symptoms or IL-2 elevation.
Kelly et al. (38)	Phase 1/2a RCT	TAK-101	Gliadin-nanoparticle tolerance induction	HLA-DQ2/8+ CeD, 18–75 years, GFD ≥6 months	PK, safety, tolerability, IFN-*γ* + cells; Enteropathy, IELs, gut-homing T cells	≥14 days	Flushing, headache, back pain. No serious AEs occurred (no differences between groups)	88% reduction in IFN-γ + cells. No Vh:Cd deterioration in TAK-101 group.
Lähdeaho et al. (39)	Double-blind RCT	AMG 714	Anti-IL-15 antibody	CeD, 18–80 years, GFD >12 months	Vh:Cd ratio change; IEL density, symptoms, antibodies	2-4 g daily for 10 weeks	Gastrointestinal symptoms (no differences between groups). Injection site reactions occurred more frequently in AMG group.	No significant Vh:Cd difference. 300 mg improved IEL density and symptoms.
Celier et al. (40)	Double-blind RCT	AMG 714	Anti-IL-15 antibody	Adults with confirmed refractory CeD type 2	Change in aberrant IEL from baseline to week 12; histological scores, patient-reported symptoms	None, GFD continued	Nasopharyngitis (42% AMG 714 vs. 11% placebo) Five serious AEs in AMG 714 group vs. one in placebo	No significant difference in primary endpoint but associated with symptom improvement
Schuppan et al. (41)	Double-blind RCT	ZED1227	Transglutaminase 2 inhibitor	HLA-DQ2/8+ CeD, 18–65 years, GFD >12 months	Vh:Cd change; IELs density, symptom scores, QoL	3 g/day for 6 weeks	Headache, nausea, vomiting, abdominal pain. No differences between groups, except for rash (3 patients, 8%) in the 100 mg group.	ZED1227 attenuated mucosal damage. 100 mg dose may improve symptoms and QoL.

RCT, Randomised Controlled Trial; CeD, Coeliac Disease; GFD, Gluten-Free Diet; Vh:Cd, Villus height to Crypt depth ratio; IEL, Intraepithelial Lymphocyte; QoL, Quality of Life; PK, Pharmacokinetics; IL, Interleukin; IFN, Interferon; AEs, Adverse Events.

### 6.1 Nexvax2

Nexvax2, was the first therapeutic vaccine created to treat CeD. It consists of synthetic peptides recognised by gluten specific CD4+ T-lymphocytes, leading to their non-reactivity to further gluten stimuli (35).

A phase 1 randomised placebo-controlled trial was initially conducted to evaluate the safety and tolerability of Nexvax2, highlighting that the vaccine did not cause changes in circulating lymphocyte subgroups and no significant changes in the villus-crypt ratio. A subsequent phase 2 study was conducted to evaluate the efficacy of Nexvax2, but it did not demonstrate any beneficial effect in lowering the levels of circulating coeliac antibodies (anti-tTG, anti-DGP), improving duodenal histology and reducing gastrointestinal symptoms (35–37). Regarding AEs, nausea and bloating were usually more frequent in Nexvax2 group (35, 36).

So far, trials on Nexvax2 have been discontinued after unsatisfactory results of phase 2 studies.

### 6.2 TAK-101

Recently, another drug used to induce immunotolerance was engineered, its name is TAK-101 and it consists of gliadin encapsulated in nanoparticles to induce tolerogenic effects (38).

By administering the drug TAK-101 intravenously instead of subcutaneously as Nexvax2, antigen-presenting cells (APCs) with tolerogenic properties in the liver and spleen are activated instead of APCs with immunogenic properties in the skin and lymph nodes. This different approach allows for the induction of an anergic state in gluten-specific T-lymphocytes, while simultaneously activating regulatory T-lymphocytes, which are crucial for achieving the desired tolerogenic effect.

In a phase 2a trial, 33 patients were randomised to TAK-101 and placebo. The number of circulating gliadin-specific IFN-gamma spot-forming T-cells in response to oral gluten challenge was reduced in TAK-101 group compared to placebo. Furthermore, this drug prevented the deterioration of villous-crypt ratio compared to placebo, even if this did not reach the statistical significance (*p* = 0.1). On the contrary, TAK-101 did not induce clinical changes and did not decrease the percentage of IELs (38).

TAK-101 was generally well tolerated, and no serious AEs occurred. Flushing, headache, back pain, were the most commonly reported AEs, with significant differences between treatment and placebo groups.

Regarding future perspectives, TAK-101 seems promising with its unique mechanism of action, but larger trials are needed to confirm preliminary results.

### 6.3 AMG 714

The monoclonal antibody AMG 714 administered by intravenous infusion exploits a different mechanism of action, namely the inhibition of IL-15 production by APCs and epithelial cells (39).

IL-15 plays a fundamental role in the activation and proliferation of lymphocytes, making CD4+ T-lymphocytes insensitive to the inhibition of regulatory T-lymphocytes and promoting the loss of tolerance to food antigens. AMG 714 did not induce statistically significant changes in the villous-crypt ratio compared with placebo, but only an improvement in lymphocyte density and clinical picture was observed. The authors therefore concluded that AMG 714 may be used beneficially in coeliac patients with persistent symptoms despite a GFD (39).

The effect of AMG 714 was also investigated for type 2 refractory coeliac disease (RCD) in a RCT, given its pathophysiological link with IL-15. After 10 weeks of AMG 714 or placebo, there was no difference between the groups in terms of histological endpoints; nevertheless, patients in the AMG 714 group showed improvement of symptoms compared to the placebo group (40). Serious AEs were reported in 5 patients (26.3%) in the AMG 714 group (pneumococcal infection, elevated transaminases, balance disorder, tuberculosis, and cerebellar syndrome). Safety profile was considered acceptable by the authors considering the severity of RCD type 2. Nasopharyngitis was also commonly reported in AMG 714 group compared to the placebo group (42%vs. 11%).

Overall, the results obtained for AMG714 were poorly satisfactory. Future developments are unlikely due to poor efficacy and concerns regarding its safety profile.

## 7 Inhibitors of tissue transglutaminase 2

### 7.1 ZED1227

ZED1227 is an orally administered small molecule tissue transglutaminase (TG2) inhibitor that selectively binds to the active form of TG2, thus preventing the formation of deamidated gliadin, its antigenic presentation resulting in gluten-induced T-cell activation (41).

In a double-blind, placebo-controlled study, ZED1227 demonstrated efficacy compared to placebo in reducing mucosal injury and preserving the villous-crypt ratio (*p*-value <0.001) in CeD patients undergoing a moderate-dose gluten challenge (3 g/daily for 6 weeks), in all proposed dosage (10, 50, 100 mg). Moreover, the effectiveness of ZED1227 has been shown to be dose dependent, with doses ranging from 50 to 100 mg exhibiting greater efficacy in preventing intestinal villous atrophy compared to 10 mg. Furthermore, 100 mg of ZED1227, was effective in inhibiting the increase of IELs consequently to gluten ingestion.

Regarding AEs, headache, nausea, vomiting were the most commonly reported, but there were no differences between groups, except for rash, which occurred in 3 patients (8%) in the 100 mg treatment group.

ZED1227 appears to be the most promising candidate drug with demonstrated dose-dependent efficacy and a good safety profile. It is likely to progress to further development and there is ongoing recruitment for a phase II, double-blind, randomised, placebo-controlled trial in coeliac patients with persistent symptoms despite a GFD (EudraCT/CTIS number 2023–506150-21).

## 8 Miscellanea

### 8.1 Probiotics

There is significant evidence that gut microbiota can influence and alter the immune system, playing an important role in maintaining a healthy state. Consequently, it is plausible that in genetically susceptible host, imbalances between microbiota and immunity could lead to the onset of a major immune-mediated inflammatory disease, including CeD (42, 43). Three randomised placebo-controlled trials have investigated the role of probiotics as an alternative to a GFD.

In a three-month double-blind, placebo-controlled randomised study, *Bifidobacterium longum* CECT7347 was found to attenuate the inflammatory effects of dysbiotic intestinal microbiota, decreasing peripheral CD3+ T lymphocytes (*p* = 0.004), slightly reducing TNF-*α* concentration (even though it was not statistically significant, *p* = 0.067), reducing the numbers of the *Bacteroides fragilis* group (*p* = 0.02) and the content of fecal IgA (*p* = 0.011) (44).

Others examined the role of VSL#3*™*, a well-known probiotic mixture used in inflammatory bowel disease, on patients with CeD. Harnett et al. randomised 42 CeD patients with only partial symptom improvement despite strict adherence to a GFD, in a group treated with VSL#3*™* and a placebo group for 12 weeks (45). Unfortunately, no significant differences were found between the two groups at the end of the treatment in bacteria, mycotoxins, or parasites composition, nor for blood urea levels or urinary organic acids.

### 8.2 Rifaximin

Rifaximin is a non-absorbable, broad spectrum antibiotic, which acts as an inhibitor of bacterial RNA synthesis and it is mainly used to treat travelers’ Diarrhoea and irritable bowel syndrome. Chang et al. conducted a single-center, double-blind, randomised, placebo-controlled study involving 50 patients to evaluate the improvement of gastrointestinal symptoms in patients with non-responsive CeD with a dose of 1,200 mg per day for 10 days of rifaximin. After randomisation, authors concluded that rifaximin did not improve symptoms in CeD patients with persistent gastrointestinal symptoms following a GFD (46).

### 8.3 Budesonide

The efficacy of budesonide for RCD is well known, on the contrary fewer were the studies about its role in acute reactions to gluten or as alternative of GFD (47, 48).

The impact of budesonide was assessed, in an *in vivo* and *in vitro* pilot study, in 20 patients randomised to GFD with or without 6 mg/day of budesonide (49).

Individuals receiving both a GFD and budesonide reported higher well-being scores, increased body weight, reduced frequency of evacuations, and decreased stool weight compared to those on a gluten-free diet. Duodenal biopsies in CeD patients and non-CeD patients were exposed *in vitro* to gliadin (0.5 mg/mL) and budesonide (10–30 μg/mL) for 3 and 24 h. *In vitro* budesonide led to a decrease in epithelial tyrosine phosphorylation and histocompatibility leucocyte antigen complex DR (HLA-DR) expression induced by gliadin-derived peptides and in cyclo-oxygenase (COX)-2 and intercellular adhesion molecule (ICAM)-1 in the *lamina propria* compared to those treated with gliadin alone (49).

Budesonide was also assessed to evaluate its effect on histological response, but no statistically significant differences were observed regarding Marsh grading and villous-height in the studies (48, 50). No major AEs occurred during the therapy with budesonide.

### 8.4 Necator americanus

Parasitic helminths may potentially regulate gut microbiota and alter the progression of inflammatory disease.

A successful small trial (12 patients) was conducted by an Australian team by inoculating subcutaneously *Necator americanus* larvae in CeD patients undergoing gluten challenge (GC), which prevented the worsening of villous trophism and symptoms (51). However, a subsequent larger (54 patients) randomised, placebo-controlled trial failed to reproduce the previous results but confirmed the protective effects on symptoms (52). However, the inoculation of *Necator americanus* larvae appeared safe, with no severe AEs occurred.

## 9 Clinical trials pitfalls

A major problem of the trials conducted so far is the heterogeneity of aspects related to the populations recruited, the endpoints and the outcomes measures. Thus, this represents a barrier to compare and generalise the results of these studies. We will briefly discuss the major pitfalls emerging from phase 2 trials conducted so far.

### 9.1 Concept of cross contamination

Cross-contamination and inadvertent gluten intake have always been a significant fear for coeliac patients to cope with. However, to define the concept of cross contamination to a GFD is very difficult, as currently no precise definition exists in the literature. It is well known that 50 mg of gluten/day for 90 days represents the minimal toxic dose for coeliac patients; on the other hand, 10 mg of gluten/day is the maximum non-toxic amount of gluten for coeliac patients (53, 54). With regard to these doses of gluten, it has been previously shown that 50 mg of gluten (equivalent to 0.05 grams of gluten) are contained in food samples that a well instructed and conscientious coeliac patient is not likely to eat by mistake. In practical terms, 50 mg of gluten are contained in a large breadcrumb, with a size of approximately 1–2 cm, if we consider that gluten is 75% of the whole protein content of wheat (55). In the trials conducted so far where gluten was administered to patients, the dose varied between 2 g (roughly equivalent to a slice of bread or a packet of crackers) and 16 g per day (roughly equivalent to a large serving of Italian pasta) (20–39, 56, 57), which is definitely a toxic dose of gluten that is very unlikely to be eaten by mistake. A recent international consensus on outcomes measures for CeD trials established that 9 g of gluten/die is the maximum amount tolerated for clinical trials to simulate normal ingestion (58), which is more or less the equivalent of 90 gr of common Italian pasta (a medium portion).

Another relevant aspect to consider is that, although inadvertent gluten intake has been repeatedly reported as a leading cause for persistent symptoms in CeD (11–13), particularly in those patients who may be supersensitive (59), it is very difficult to properly ascertain its causative role in clinical practice. In this regard, a recent study by our group showed that minimal and inadvertent ingestion of gluten in coeliac patients who had been correctly instructed on how to follow a GFD is likely to have no role on triggering intestinal symptoms (60).

## 10 Study population

Heterogeneity of coeliac patients enrolled in the trials is another point to critically consider. The vast majority of trials enrolled adult coeliac patients with confirmed diagnosis based on both serology and duodenal histology, who have been on a GFD for at least 6–12 months even without evidence of histological response to a GFD at time of enrolment. Additionally, the majority of them lacked a ‘baseline biopsy’ before recruitment into the trials, and only some of them performed a follow-up duodenal biopsy in the 6 months prior to enrolment due to clinical reasons (21, 32, 36, 57).

Adequate knowledge of the GFD is a crucial requirement for coeliac patients, and several reports highlight the association between a comprehensive knowledge of gluten-free living and a better adherence to a GFD (61–63). Consequently, knowledge about a GFD should be assessed before enrolment in a clinical trial, but unfortunately this has not been systematically done and was limited to self-reported adherence.

Furthermore, HLA DQ2.5 typing was also used as a diagnostic criterion for many trials (21, 35–37), which potentially represents a limit towards excluding other patients expressing HLA-DQ8 molecules or other rarer haplotypes such as HLA-DQ2.2 and HLA-DQ7.5.

Special subgroups such as CeD patients with persistent symptoms and refractory CeD were only rarely included and evaluated (23, 40), unfortunately with unsuccessful results.

Finally, all the trials conducted so far involved adult coeliac patients only and no data on pediatric populations are available. This is an important aspect to be considered in the future, also based on recent EU regulations.

### 10.1 Gluten-challenge (dose, duration, vehicle)

A major factor to consider in the evaluation of drug efficacy is the administration of a gluten challenge. In fact, dose, vehicle of gluten administration and duration of gluten-challenge in trials have not been standardised so far. As previously mentioned, GC dose varied between 2 g and 16 g per day (20–39, 56, 57). This aspect is even more challenging if we consider that also in clinical practice diagnostic gluten-challenge is complex to perform. A recent ESPGHAN position paper (64) provides guidance on how to perform GC in children, although this is mainly based on expert opinion, whereas in adults no guidelines provide guidance for gluten challenge (65–67). Table 5 summarises the main concerns related to GC in clinical trials.

**Table 5:** Phase-2 studies performing gluten challenge.

Study	GC dose	GC duration	GC vehicle	GFD duration before study	Run-in period	Baseline histology
Goel et al. (35)	9 g/day for days 1–3; then 9 g/day for days 8–10	3 + 3 days	Cookies (3 g gluten each)	>12 months	No	Yes
Truitt et al. (36)	6 g	Single bolus	10 g vital wheat gluten flour in water	>12 months	No	No
Tye-Din et al. (37)	10 g bolus	Single dose	10 g vital wheat	>12 months	No	Yes
Schuppan et al. (41)	3 g/day	6 weeks	1 biscuit/day	>12 months	No	Yes
Kelly et al. (38)	12 g/day for 3 days, then 6 g/day for 11 days	14 days	NA	>6 months	No	Yes
Lähdeaho et al. (39)	2-4 g/day	10 weeks	Two cookies/day (Finnish rusks or double-baked cake breads)	>12 months	No	Yes
Paterson et al. (31)	2.5 g bolus	Single dose	Pudding with 2.5 g amygluten 160 powder	>6 months	No	Yes
Leffler et al. (65)	2.4 g/day	14 days	Capsules (amgluten 160 powder)	>6 months	No	No
Kelly et al. (32)	2.7 g/day	6 weeks	Capsules (450 mg gluten each)	>6 months	Yes, 7-days placebo	No
Tye-Din et al. (21)	16 g/day	3 days	Flour slurry mixed with orange juice or soy milk	>8 weeks	No	No
Lähdeaho et al. (22)	2 g/day	6 weeks	Breadcrumb	>12 months	No	Yes
Murray et al. (24)	2 g/day	6 weeks	NA	>12 months	Yes, 14-days placebo	Yes
Tack et al. (27)	7 g/day	2 weeks	Toast	>12 months	Yes, 14-days placebo	Yes

GC, Gluten Challenge; GFD, Gluten-Free Diet; NA, Not AvailableLEGENDS.

Run-in periods are useful to reduce drop-outs from a trial (68); however, run-in periods were introduced only in 5 trials (24, 27, 32, 36, 37). Recently, run-in periods have been suggested for trials contemplating gluten-challenge in order to increase compliance and reduce confounding in the evaluation of symptomatic response (58).

The length of GC varied greatly among studies, ranging from a single bolus dose to up to 10 weeks in one case (31, 37, 39). Furthermore, the type of gluten vehicle was not standardised and several different methods of administration were used, such as capsules but also baked products such as cookies, bread and biscuits that may be rich in fermentable oligo-, di-, and monosaccharides and polyols (FODMAP) (32, 35, 39, 40, 57), which are known to trigger symptoms in IBS and also in coeliac patients on a GFD (69, 70).

Additionally, the adherence to GC in the trials was not extensively evaluated, neither with specific questionnaires nor with objective tools such as GIP (24, 28). Lastly, the influence of the so called ‘trial effect’ on patients enrolled in trials should also be considered, as this may lead patients to improve their adherence to the GFD, potentially confounding the beneficial effect of the drug compared to controls (23).

### 10.2 Histological outcomes measures

The precise definition of histological recovery is a mandatory outcome to establish before starting a trial. This concept is challenging also in clinical practice, as many parameters should be considered such as the patchiness of duodenal lesions, the amount of time required for healing, the histological criteria adopted. So far, histology has been the primary endpoint of 5 trials (22–24, 39, 41) and this was effectively met only in one (41). Moreover, different methods (villous height to crypt depth (Vh:Cd) ratio, lactulose-to-mannitol (LAMA) ratio and densities of IELs) have been used to evaluate histological changes, which makes it difficult to compare the results and inevitably introduces an observer variability. Indeed, LAMA is not specific for CeD, but it only provides an indirect and less appropriate measure of histological damage by assessing intestinal permeability (31, 32, 56, 57). According to a recent consensus, a Vh:Cd ratio ≥ 2.5 or ≥ 3 or Marsh 1 lesions were considered necessary criteria to enter a trial where gluten challenge is performed in order to avoid the ethical concerns related to offering gluten to patients with persistent villous atrophy (58).

### 10.3 Inclusion of patient related outcomes

The use of PROs as trial endpoints has been gaining importance over the last decade, due to their extensive application in pharmacological trials, particular those related to inflammatory bowel disease and functional gastro-intestinal disorders (71–73).

PROs provide measures of patients’ QOL and assess how objective clinical effects alter the subjective sphere and viceversa. Indeed, patients’ clinical characteristics such as anxiety, resilience and hyper-vigilance could potentially skew the results, contaminate trial’s endpoints and change symptoms perception (74–76). Furthermore, PROs promote a more patient-centered evaluation and regulatory agencies such as the European Medicine Agency and the Food and Drugs Administration have also recognised their significance.

Few CeD trials have investigated PRO (37, 56), but their inclusion is desirable in future trials as suggested by a recent international consensus (58).

## 11 Considerations on efficacy of alternative pharmacological drugs

This review has summarised the current evidence about molecules evaluated in phase II trials in the last two decades, which may potentially support/replace the GFD in coeliac patients. The pursuit of an alternative, non-pharmacological therapy to GFD is highly requested by patients and industry and could represent a significant improvement in all instances where conventional therapy alone is insufficient. Although the development of alternative therapies has spanned over two decades, with varying degrees of industry interest and investment, several factors have contributed to the slow progress, including the complexity of the disease mechanism, challenges in trials design, and the high bar set by the effectiveness of the GFD.

In fact, so far, none among the proposed molecules has yet demonstrated a significant efficacy, particularly in the prevention of gluten-induced histological damage. Indeed, promising preliminary phase-II results have been observed only with ZED1227, a transglutaminase-2 inhibitor, whose administration has reduced gluten-induced mucosal damage, demonstrating a good safety profile (41). However, the small sample size precludes to give definitive results. This molecule is currently undergoing a phase IIb trial, under the name rebranded in TAK-227 (EudraCT number 2020–004612-97) (77).

The remaining therapies aiming to induce immune tolerance to gluten have failed to meet the primary endpoint represented by the prevention of the histological damage, although a minimal positive effect on the prevention of gluten-induced damage has been shown for TAK-101 (38). For this reason, a new trial is currently ongoing (NCT04530123) (78).

Currently, phase 2 studies on glutenases are yielding disappointing results regarding their effectiveness, particularly in the prevention of mucosal damage after gluten challenge. Therefore, their potential target population may be represented by patients with ongoing symptoms despite a GFD and no histological damage.

Larazotide held high interest in the past, but now it is clear that it is unable to prevent mucosal damage (31, 32, 56, 57). Nevertheless, it may be still considered for symptoms control in the absence of mucosal damage/organic disorders.

In conclusion, the possibility to develop alternative or supportive therapies to a GFD still remains a priority in the research agenda in this field. Identification of specific subgroups of patients and meaningful endpoints together with uniformity in the trial methodology are major areas to implement in the future.
